# Evaluation of Bonding Shear Performance of Ultra-High-Performance Concrete with Increase in Delay in Formation of Cold Joints

**DOI:** 10.3390/ma9050362

**Published:** 2016-05-12

**Authors:** Han-Seung Lee, Hyun-O Jang, Keun-Hee Cho

**Affiliations:** 1 Department of Architecture Engineering, Hanyang University, 55, Hanyangdaehak-ro, Sangrok-gu, Ansan-si, Gyeonggi-do 15588, Korea; ercleehs@hanyang.ac.kr; 2 Structural Engineering Research Institute, Korea Institute of Civil Engineering and Building Technology, 283, Goyangdae-ro, Ilsanseo-gu, Goyang-si, Gyeonggi-do 10223, Korea; kcho@kict.re.kr

**Keywords:** ultra-high-performance concrete (UHPC), cold joint, bond strength, direct shear test, delay, SEM, XRD

## Abstract

This study set out to derive the optimal conditions for ensuring the monolithicity of ultra-high-performance concrete (UHPC). Direct shear tests were performed to examine the influence on the bonding shear performance. The experimental variables included tamping and delay, which were set to 0, 15, 30, and 60 min. SEM and XRD analyses of the microstructure and composition were performed. The direct shear tests showed that the bonding shear strength was enhanced by the addition of tamping. For the normal-strength concrete (NSC), it is thought that a monolithicity of around 95% can be attained with a cold joint formation delay up to 60 min. In contrast, while the normalized bonding shear strength reduction of UHPC with a delay of 15 min was the lowest at around 8%, a dramatic degradation in the bonding shear performance was observed after 15 min. XRD analyses of the middle and surface sections revealed the composition of the thin film formed at the surface of the UHPC and, as a result, the main component appeared to be SiO_2_, which is believed to be a result of the rising of the SiO_2_-based filler, used as an admixture in this study, towards the surface, due to its low specific gravity.

## 1. Introduction

As a construction material, concrete has, over a very long period, become essential to the infrastructure of modern society. Advances in economy, society, and culture have created a large demand for social infrastructure such as bridges, roads, railways, harbors, and airports. Moreover, given the growth in the construction of large-scale, high-rise, and long-span structures, there is a need for high-performance concrete, which offers enhanced performance in terms of its workability, strength, and durability, relative to previous materials [1,2]. As such, the application of ultra-high-strength concrete with a strength of over 150 MPa has been realized through the optimization of the aggregate composition based on the theory of optimum micro-fillers. Furthermore, this has led to the development of high-toughness, ultra-high-performance concrete (UHPC), which exhibits both high strength, and high ductility, by adding steel fibers to compensate for the brittleness of the concrete [3,4,5,6,7,8]. However, it is known that the fast setting of UHPC due to its low water-binder ratio (*W*/*B*) and the relatively small amount of bleeding affect the bonding shear performance [9,10]. If this type of UHPC were to be utilized for construction projects and the pouring of the concrete was to pause temporarily, due to the failure of the construction equipment or a delivery delay, cold joints would occur, where the monolithicity between the concrete poured previously and that poured later is not ensured. This produces an imperfect bonding surface, which is prone to becoming a structural or material vulnerability. In particular, cold joints occur arbitrarily in unexpected locations, and lead to cracking and leaking upon the occurrence of shrinkage as the concrete hydrates, as well as the corrosion of the reinforcement bars, which can lead to the degradation of the durability of the concrete. Rathi and Kolase performed split tension tests with respect to the delays for concrete of normal and different strengths [11]. Moreover, Xiao *et al.* evaluated the shear behavior of recycled aggregate concrete with respect to the state of the joint surface [12]. Martin and Rivard investigated the effect of freezing/thawing on the shear strength of concrete cold joint [13]. However, there has been a minimal research addressing UHPC that considers the delay and characteristics of the cold joints.

UHPC exhibits its optimal structural performance and durability only when monolithicity is ensured. To ensure the monolithicity of UHPC despite the occurrence of cold joints, it is necessary to account for its characteristics and, at the same time, to quantitatively evaluate the bonding shear performance. In particular, it is necessary to examine the effect of any delay on the degradation of the bonding shear performance.

Therefore, this study set out to derive the optimal conditions for ensuring the bonding shear performance considering the delay leading to the occurrence of cold joints during the pouring of UHPC (180 MPa). Also, to determine the cause of the degradation of bonding shear performance, NSC (24 MPa) is set for control group. The difference of bond shear performance between UHPC and NSC is determined by performing a direct shear test and microanalysis of SEM and XRD at three days and 28 days.

## 2. Experimental Plan

### 2.1. Overview of Experiment

In this study, a direct shear test was performed to evaluate the bonding shear performance of the cold joints that occur as a result of delays during the pouring of concrete. Moreover, to derive the mechanism influencing the bonding shear performance, visual, SEM and XRD analyses were performed for different delays.

The experimental factors and their respective levels are listed in Table 1, and the experimental variables are listed in Table 2.

**Figure 1:**
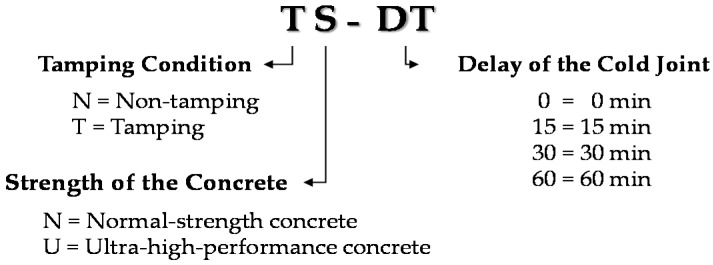
Overview of specimen nomenclature.

The naming of the specimens was based on the tamping of the concrete, strength of the concrete, and delay. The specimen nomenclature is shown in Figure 1. Taking the NN-0 specimen in Table 2 as an example, the first N represents the tamping condition, the second N represents the strength of the concrete, and the last number represents the delay of the pouring.

**Figure 2:**
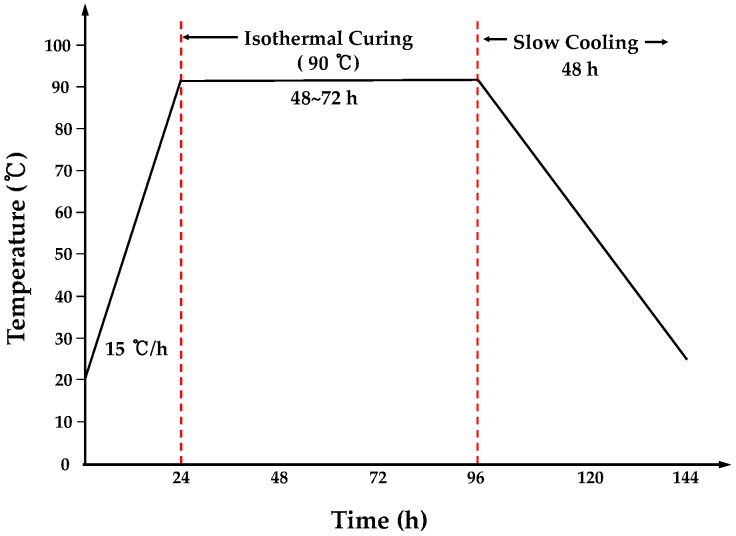
Heating cycle of high-temperature steam curing.

The curing method applied to the specimens examined in the direct shear test involved removing the specimens one day after pouring, and subsequently allowing them to high-temperature steam for six days, as shown in Figure 2. Moreover, the curing method applied to the specimens subjected to microstructure analysis involved air drying for 28 days (temperature: 20 °C, relative humidity: 60%).

### 2.2. Materials and Mix Ratio

Type-1 Ordinary Portland Cement (OPC) with a density of 3.15 g/cm^3^ was used, in accordance with KS L 5201 [14]. The chemical composition of the binder is listed in Table 3. As such, the *W*/*B* ratio of the NSC consisting of sand, gravel, and cement was 0.4 and chemical composition of aggregates are listed in Table 4. Moreover, the W/B ratio of the steel-fiber-reinforced UHPC consisting only of sand was 0.14. The Australian silica sand (76% SiO_2_ content and 0.5 mm average particle diameter) was used and its specific gravity is 2.65. Additionally, polycarboxylate-based high-performance air-entraining admixture is used for fluidity of concrete. The properties of admixture are listed in Table 5. The concrete mix proportion is listed in Table 6.

### 2.3. Experimental Methods

#### 2.3.1. Manufacturing of the Specimens

**Figure 3:**
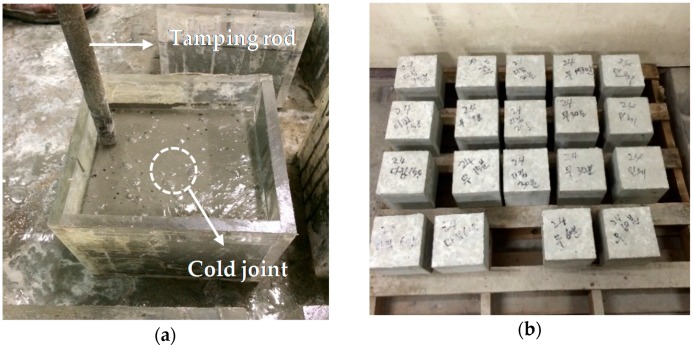
Manufacturing of specimens for direct shear test: (**a**) tamping of the specimen, prior to the second pouring; and (**b**) specimens after removal.

The specimens were manufactured using a 150 mm × 150 mm × 150 mm acrylic mold, as shown in Figure 3.

As shown in Figure 3a, 150 mm × 150 mm × 100 mm specimens were manufactured by pouring the first-stage concrete, followed by the second-stage pouring, in the case of the non-tamped specimens, 0, 15, 30, and 60 min after. Tamped 150 mm × 150 mm × 150 mm specimens were manufactured by tamping the first-stage concrete with a tamping rod, and then pouring the second stage concrete.

#### 2.3.2. Material Characteristics of Concrete

A slump flow test was conducted in accordance with KS F 2594 [15]. Moreover, NSC and UHPC circular specimens with dimensions of Ø100 × 200 mm and Ø50 × 100 mm, respectively, were manufactured in accordance with KS F 2403 [16]. Air dry curing was performed by using the thermo-hygrostat under the condition of 20 ± 2 °C, 60% ± 2% RH, for both seven and 28 days. The compressive strength was measured using a universal testing machine (UTM) in accordance with KS F 2405 [17], and the average was taken for each level for the three specimens.

#### 2.3.3. Direct Shear Test

Various methods have been suggested for evaluating the bonding shear performance with respect the occurrence of cold joints [18,19]. In general, the bonding shear performance can be evaluated based on the bonding shear strength. This can be classified into a slant shear test and a direct shear test. However, it has been noted that the slant shear test, as specified in BS6319: Part 4 [20], suffers a disadvantage in that the failure varies with the angle of the bonding interface, since any stress in the interface combines compressive stress and shear stress [21].

**Figure 4:**
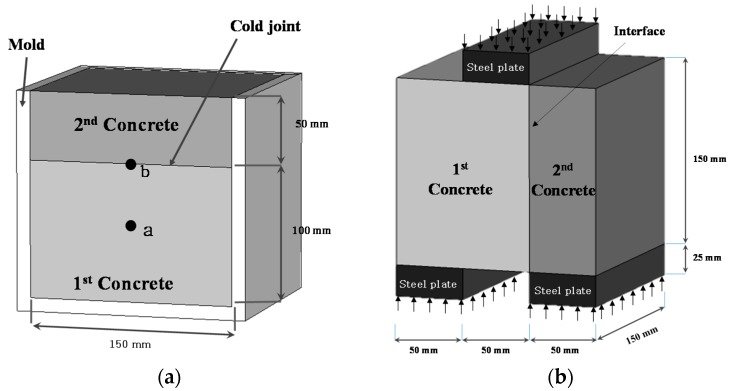
Overview of direct shear test: (**a**) locations of sample collection for microstructure analysis; and (**b**) direct shear test method and specimen size.

As such, in this study, from the material’s viewpoint the evaluation of bonding shear performance was performed according to different delays for the cold joint by applying a direct shear test [22]. In Figure 4b, direct shear test progressed by placing 50 mm × 150 mm × 25 mm steel plate on the center and each end of the sample. The shear bond strength was measured by using 200 ton UTM (Universal Testing Machine) under the condition loading rate of 0.01 mm/s.

The bonding shear strength was computed by dividing the maximum load by the geometrical surface area, as given by Equation (1), below [23,24]:

(1) $$f_{b}=\;\frac{F}{2A}$$

where, $f_{b}$ is the bonding shear strength (MPa); *F* is the maximum load (N); and *A* is the shear bonding area (${\text{mm}}^{2}$).

#### 2.3.4. Analysis Using Scanning Electron Microscopy (SEM)

The microstructures of locations a (middle section) and b (cold joint region), locations identified in Figure 4a, were analyzed using SEM. The UHPC and NSC specimens were manufactured with a concrete mix that does not contain fine or coarse aggregates.

#### 2.3.5. X-ray Diffraction (XRD) Composition Analysis

XRD composition analysis was performed for locations a (middle section) and b (cold joint region), locations identified in Figure 4a. The UHPC and NSC specimens were of concrete mix with no aggregates. The XRD device was the RIGAKU D/MAX-2500/PC. The measurement range was 5°–70° (2θ) and the measurement was performed under the condition of Cu (40 kV, 100 mA).

## 3. Experimental Results and Analysis

### 3.1. Material Characteristics of Concrete

To identify the material properties of NSC and UHPC, slump flow and compressive strength tests were conducted. The results are listed in Table 7.

From the flowability measurements, it was observed that the slump of NSC is 163 mm and that of UHPC is 680 mm. These satisfy both the target slump of NSC and the target slump flow of UHPC, which were set to 150 ± 50 mm and 700 ± 50 mm, respectively, for this study. Moreover, from the compression test, the compressive strengths of NSC and UHPC after seven days of steam curing were measured to be 26 MPa and 174 MPa, respectively. Furthermore, the compressive strengths of NSC and UHPC after 28 days of air drying were measured at 25 MPa and 127 MPa, respectively.

### 3.2. Evaluation of Bonding Shear Performance with Variations in Delay of Cold Joint Occurrence

#### 3.2.1. Change in Bonding Shear Strength with Tamping and Delay

**Figure 5:**
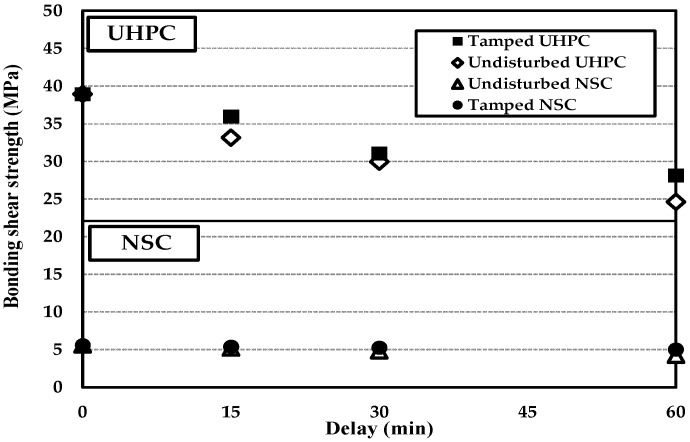
Comparison of bonding shear strengths of NSC and UHPC with tamping and varying delays.

The results of the direct shear test are listed in Table 8. Moreover, Figure 5 illustrates the change in the bonding shear strength with the delay.

The bonding shear strengths of NSC and UHPC appeared to be in the order of 0 min > 15 min > 30 min > 60 min, regardless of whether tamping was applied. The bonding shear strengths of NN-0 and NU-0 were measured and found to be 5.6 MPa and 38.9 MPa, respectively. Subsequently, the bonding shear strength exhibited a tendency to gradually decrease as the delay increased. Moreover, in the tamped cases, the bonding shear strengths of NSC and UHPC tended to increase by around 5% and 7%, respectively.

**Figure 6:**
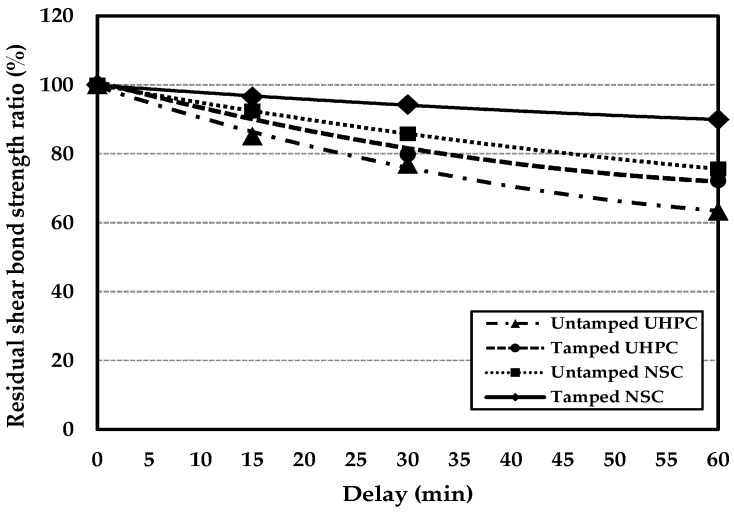
Comparison of ratios of decrease in the bonding shear strengths of NSC and UHPC with tamping and increasing delay.

Figure 6 shows the normalized bonding shear strength reduction with respect to the delay. Compared to NN-0, NSC specimens NN-15 and NN-30 exhibited ratios of decrease of around 8% and 14%, respectively. Moreover, NN-60 exhibited a normalized bonding shear strength reduction of around 24%. However, the tamped specimen, TN-60, exhibited a normalized bonding shear strength reduction of around 10%. This is attributed to the formation of bleed water at the pouring surface of the first-stage concrete during the 60-min delay after the pouring, which results in a sound bonding shear performance.

With the UHPC specimens, the ratios of decrease in the bonding shear strengths of NU-15 and NU-30 appeared to be around 15% and 23%, respectively, relative to NU-0. Moreover, TU-15 exhibited a normalized bonding shear strength reduction of around 8%, indicating the best bonding shear performance. However, the ratios of decrease in the bonding shear strengths of TU-30 and TU-60 appeared to be around 20%–28%, indicating that the bonding shear performance of the UHPC dramatically degrades with a delay in excess of 15 min. It is believed that the bonding shear performance degrades as a result of no bleed water being generated at the pouring surface of the UHPC, due to its characteristically low *W*/*B* ratio. Moreover, after 15 min, a thin film had formed at the pouring surface. It is thought that this property of the surface affects the bonding shear performance of cold joints. 

Therefore, the optimal normalized bonding shear strength reduction is within 10%. Also, the optimal delay for tamped NSC is 15 min. Furthermore, it is believed that the monolithicities of tamped NSC and UHPC can be ensured by adopting delays of up to 60 min and 15 min, respectively.

#### 3.2.2. Failure Modes

**Figure 7:**
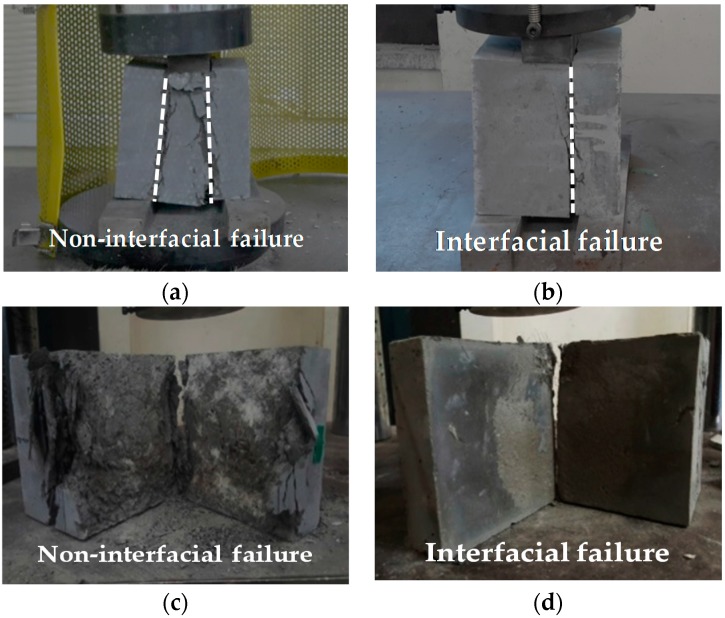
Failure modes: (**a**) non-interfacial failure; (**b**) interfacial failure; (**c**) cross-section of non-interfacial failure; and (**d**) cross-section of interfacial failure.

Figure 7 shows the failure modes of the specimens after a direct shear test. In this study, the failure modes were evaluated for Interfacial Failure and Non-Interfacial Failure. While the interfacial failure is a complete de-bonding at the transition zone, the non-interfacial failure is a complete substratum failure with good interface. The results are listed in Table 9.

NSC specimens NN-30 and NN-60 exhibited interfacial failure modes. Additionally, all the other NSC specimens exhibited non-interfacial failure modes. In contrast, UHPC specimens except NU-0 and TU-15 showed interfacial destruction modes. Moreover, the non-interfacial failure patterns were similar to those shown in Figure 7c, while the interfacial failure patterns were similar to those shown in Figure 7d. Therefore, in case of NSC and UHPC failure mode, it is possible to distinguish between the interfacial and non-interfacial destruction for a normalized bonding shear strength reduction of around 10%. However, since the tamped UHPC specimens for which the delay was more than 15 min exhibited interfacial destruction, it is thought that their monolithicity would not be ensured. As such, for the UHPC, it is deemed necessary to apply physical or chemical interfacial treatments on the shear bonding surface if the delay between pours is greater than 15 min. Moreover, it is thought that there is a need to determine the characteristics of the film formed on the surface 15 min after the UHPC is poured.

### 3.3. Microstructure Analysis of NSC and UHPC

#### 3.3.1. Temporal Changes in the Surface of Cold Joint Region with Delay 

**Figure 8:**
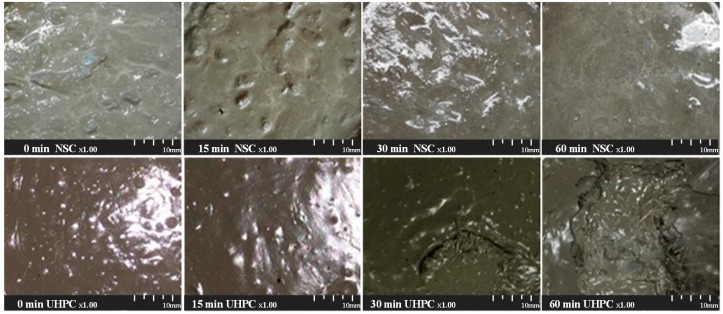
Temporal change in surface of cold joint regions of NSC and UHPC with delay.

Figure 8 shows the temporal change in surface of cold joint regions of NSC and UHPC with delay. It can be seen that NSC did not exhibit any significant changes until 15 min had elapsed. However, bleed water was observed rising to the surface at 30 and 60 min. Meanwhile, a thin film was formed at the surface of UHPC once 15 min had elapsed. Moreover, it was observed that the thickness of the film increases as the elapsed time increases, as observed after 30 and 60 min. As such, the surface films formed on the specimens after delays of 30 and 60 min were manually removed, so that the non-set surfaces could be observed.

An analysis of the properties of the pouring surfaces of the NSC and UHPC revealed that the UHPC exhibits a completely different surface pattern to that of NSC. It is thought that sound bonding shear performance can be ensured for NSC, owing to the formation of the bleed water on the surface after a delay of 30 min and more. As such, it is believed that the bonding shear performance of UHPC falls dramatically due to the film formed on the surface after a delay of as little as 15 min.

#### 3.3.2. SEM Image Analysis of NSC and UHPC

To identify the mechanism influencing the characteristics of UHPC in terms of its bonding shear strength, the middle section and the surface of a cold joint were imaged using SEM, in order to analyze the hydration products and the microstructure.

**Figure 9:**
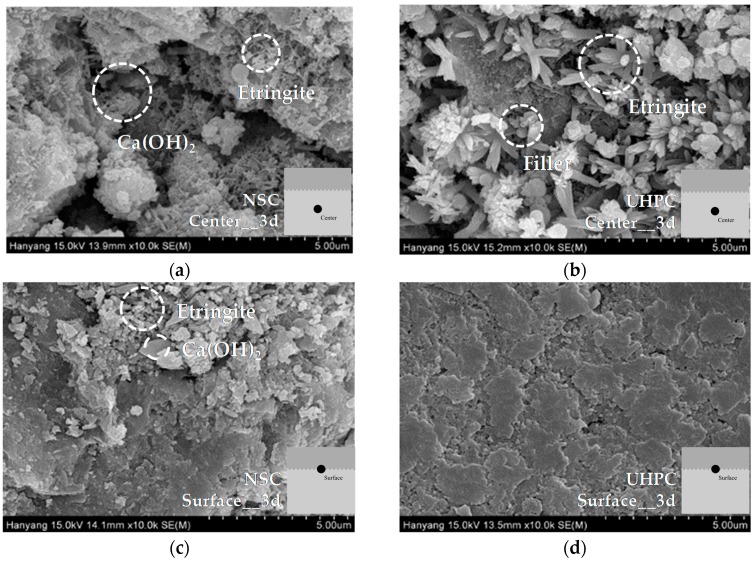
SEM analysis of middle sections and cold joint surfaces of NSC and UHPC (aged for three days): (**a**) middle section of NSC; (**b**) middle section of UHPC; (**c**) cold joint surface of NSC; and (**d**) cold joint surface of UHPC.

SEM analysis results for specimens that had been aged for three days are shown in Figure 9. In the NSC, ettringite and Ca(OH)_2_ crystals were formed in the middle section and at the cold joint surface. In contrast, in the UHPC, again aged for three days, a large amount of cement hydration product was observed in the middle section. Moreover, spherical fillers were observed in the air gaps. It is believed that a filler effect will occur due to the existence of these fillers in the air gaps. In the cold joint surface of the UHPC, initial hydration products such as ettringite were not observed, unlike in the middle section, and it was found that a thin film was formed. As a result of the formation of this film, the surface of the UHPC appeared relatively smooth and uniform.

**Figure 10:**
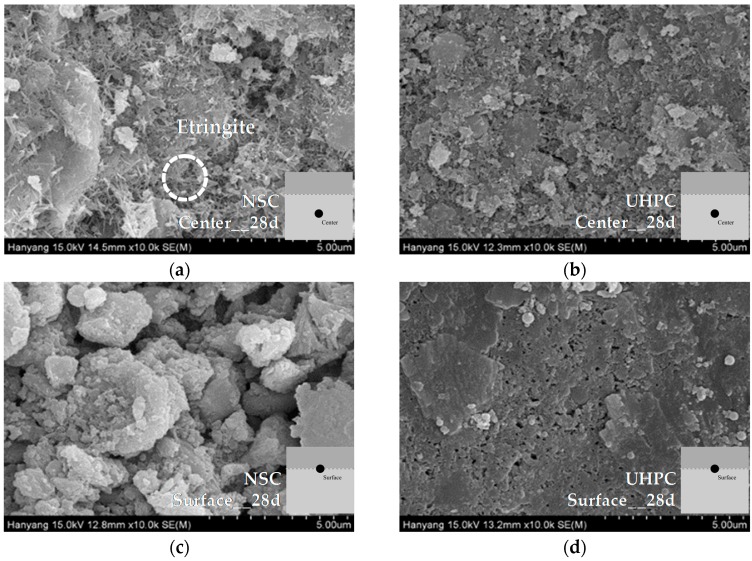
SEM analysis of middle sections and cold joint surfaces of NSC and UHPC (aged for 28 days): (**a**) middle section of NSC; (**b**) middle section of UHPC; (**c**) cold joint surface of NSC; and (**d**) cold joint surface of UHPC.

SEM analysis results for specimens aged for 28 days are shown in Figure 10. In the middle section of the NSC, more cement hydration products including ettringite and Ca(OH)_2_ were formed. Moreover, at the cold joint surface of the NSC, it was observed that the amount of ettringite decreased, and that the air gaps become denser owing to the cement matrix and Ca(OH)_2_. In the UHPC, the film formed on the cold joint surface was observed to be smoother after aging for 28 days. Moreover, a spherical crystalline structure was observed, which is speculated to be filler. This is assumed to be the cause of the degradation of the bonding shear performance of the UHPC. While the amount of ettringite in the middle section of the UHPC decreased greatly, significantly greater quantities of other hydration products were formed. Moreover, it was observed that the fillers fill the air gaps between the hydration products, and that the space between the hydration products becomes significantly denser relative to the specimen that has been aged for three days. 

#### 3.3.3. XRD Analysis of NSC and UHPC

**Figure 11:**
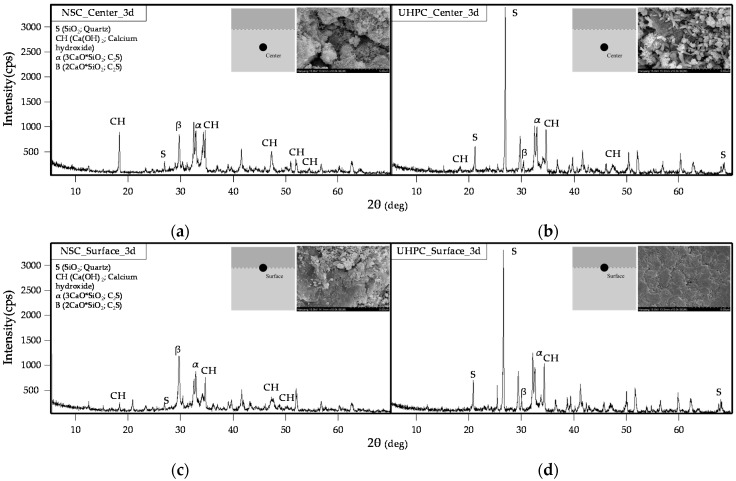
XRD analysis of middle sections and cold joint surfaces of NSC and UHPC (aged for three days): (**a**) middle section of NSC; (**b**) middle section of UHPC; (**c**) cold joint surface of NSC; and (**d**) cold joint surface of UHPC.

Figure 11 shows the results of an XRD analysis of the middle sections and cold joint surfaces of the NSC and UHPC that had been aged for three days. The XRD pattern for the NSC shows that cement minerals and Ca(OH)_2_ are the main components. Moreover, while Ca(OH)_2_ is observed in both the middle section and at the cold joint surface of the NSC that has been aged for three days, the diffraction intensity of Ca(OH)_2_ appears to be larger and more distinct in the middle section than at the cold joint surface.

**Figure 12:**
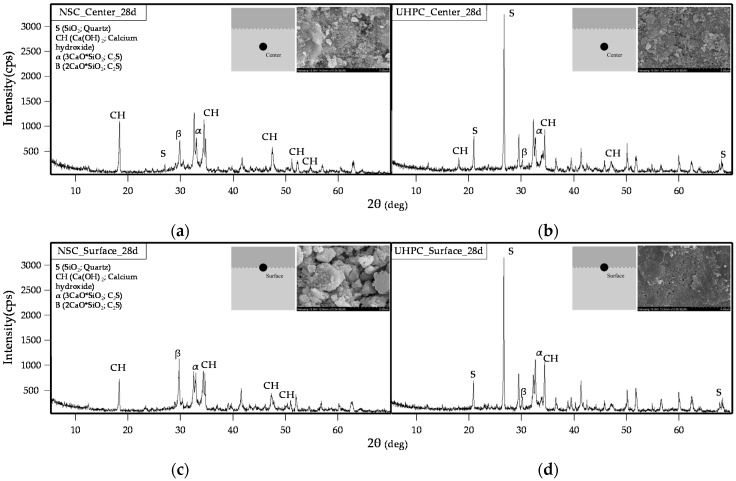
XRD analysis of middle sections and cold joint surfaces of NSC and UHPC (aged for 28 days): (**a**) middle section of NSC; (**b**) middle section of UHPC; (**c**) cold joint surface of NSC; and (**d**) cold joint surface of UHPC.

Figure 12 shows the results of an XRD analysis of the middle sections and cold joint surfaces of NSC and UHPC that had been aged for 28 days. For the NSC, the Ca(OH)_2_ content increased in the middle section and at the cold joint surface. However, a comparison of the patterns for the middle section and the surface of the NSC reveals that the diffraction intensity of the Ca(OH)_2_ is larger in the middle section than at the cold joint surface. In contrast, a comparison of the XRD patterns of the middle section and cold joint surface of the UHPC showed that the main component is SiO_2_. Moreover, relative to NSC, the diffraction intensity of Ca(OH)_2_ was observed to be lower. This can be attributed to the lack of space in which Ca(OH)_2_ crystals can form due to the extremely dense microstructure of the UHPC, as observed in a previous work [20]. A change was observed in the middle section of the UHPC, where the amount of clinker mineral decreased but that of Ca(OH)_2_ increased. In contrast, no temporal change in the pattern or the diffraction intensity was observed at the cold joint surface.

Therefore, the filler constitutes the greatest proportion in terms of bulk density ratio in the binder used in the UHPC in this study, excluding the cement. The filler has a SiO₂ content of more consists of more than 99%, and has a density of 2.60 g/cm^3^. It is thought that this filler rises to the surface of the UHPC, owing to its low specific gravity. As such, it is believed that the film formed on the pouring surface of the UHPC consists of SiO₂ crystalline structures, and that this is the cause of the degradation of the bonding shear performance.

## 4. Conclusions

From the evaluation of bonding shear performance of UHPC with respect to the time of the cold joint occurrence, the following conclusions can be drawn:

- Tamped NSC shows around a 5% increase in the bonding shear strength relative to the non-tamped NSC, and around a 5% decrease in the bonding shear strength with a delay of up to 60 min. As such, it is believed that tamping can lead to more than 95% monolithicity provided the cold joint is formed within 60 min.
- The bonding shear strength of UHPC showed a tendency to gradually decrease as the delay until the cold joint formed increased, regardless of whether tamping was performed. Tamping resulted in an enhancement of the bonding shear performance of about 7%. Moreover, a delay of 15 min resulted in a decrease in the bonding shear strength of about 8%, corresponding to indicating the best bonding shear performance.
- An analysis of the failure mode after direct shear tests revealed that the interfacial and non-interfacial failure modes could be distinguished upon a reduction in the normalized bonding shear strength of around 10%. As such, the optimal cold joint delay was found to be 60 min for tamped NSC, and 15 min for tamped UHPC. Moreover, for non-tamped cases, the optimal condition was 15 min for NSC, and it is believed that physical or chemical interfacial treatment of the bonding surface is necessary for UHPC upon the occurrence of any delay.
- From a visual observation of the poured surface of UHPC with respect to the delay of the cold joint, it was found that a thin film forms at the surface once the delay reaches 15 min, with the surface film becoming thicker once the delay reaches 60 min. Therefore, it is believed that the degradation of the bonding shear performance is due to the formation of the film at the surface.
- A SEM analysis of the middle sections and cold joint surfaces of the specimens revealed that the film of the layer structure was observed in the cold joint surface of UHPC after aging for three days, and no initial hydration products were observed. Moreover, no significant change was observed in the cold joint surface of a specimen that had been aged for 28 days, compared to that which had been aged for three days.

An XRD composition analysis of the cold joint surface of a specimen that had been aged for 28 days did not reveal any difference to that which had been aged for three days. Moreover, the main component of the cold joint surface of the UHPC appeared to be SiO₂. Therefore, it is believed that the film formed at the cold joint of UHPC consists of SiO₂ crystalline structures, which is thought to be the cause of the degradation of bonding shear performance.

## Figures and Tables

**Table 1:** Experimental factors and levels.

Experimental Factor	Levels	Number of Levels
Strength of Concrete (Proportioning Strength)	NSC ^1^ (30 MPa), UHPC (180 MPa)	2
Tamping Condition	Tamping, Non-tamping	2
Delay of the Cold Joint	0, 15, 30, and 60 min	4

^1^ Normal-strength concrete.

**Table 2:** Experimental variables.

No.	Specimen Name	1st Con’c	2nd Con’c	Tamping Condition	Delay
1	NN-0	30	-	-	0
2	NN-15	30	30	×	15
3	NN-30	30	30	×	30
4	NN-60	30	30	×	60
5	TN-15	30	30	○	15
6	TN-30	30	30	○	30
7	TN-60	30	30	○	60
8	NU-0	180	-	-	0
9	NU-15	180	180	×	15
10	NU-30	180	180	×	30
11	NU-60	180	180	×	60
12	TU-15	180	180	○	15
13	TU-30	180	180	○	30
14	TU-60	180	180	○	60

**Table 3:** Chemical composition of binder.

Division	SiO_2_ (%)	Al_2_O_3_ (%)	MgO (%)	TiO_2_ (%)	SO_3_ (%)	CaO (%)	Fe_2_O_3_ (%)	Na_2_O (%)	K_2_O (%)	f-CaO (%)	*etc.* (%)	Ig.loss (%)
OPC	19.47	5.24	3.72	-	2.49	61.8	2.69	0.18	0.87	-	0.94	2.6
F ^1^	99.47	0.4	0.009	0.04	-	0.01	0.5	0.008	0.006	-	-	-
EA ^2^	1.00	16.1	-	-	27.5	52.8	0.8	-	-	49.8	-	-
Zr ^3^	96.00	0.25	0.1	-	-	0.38	0.12	-	-	-	-	-
SRA ^4^	29.42	0.17	0.06	-	-	1.39	0.10	-	0.03	-	-	-

^1^ Filler; ^2^ Expansive admixture; ^3^ Zirconia silica fume; ^4^ Shrinkage reducing agent.

**Table 4:** Physical properties of aggregates.

Division	Fineness Modulus	Density (g/cm^3^)	Ratio of Absolute Volume (%)	Water Absorption Ratio (%)	Unit Volume Weight (kg/m^3^)
Sand	3.04	2.59	54.3	0.81	1406
Gravel	6.88	2.60	58.5	0.63	1521

**Table 5:** Properties of admixture.

Division	Density (g/cm^3^)	pH	Alkali Content (%)	Chloride Content (%)	Appearance
AE ^1^	1.05	6.5 ± 2.0	0.81	below 0.01	Light brown liquid

^1^ Air entraining and high-range water reducing agent.

**Table 6:** Concrete mix proportions.

Division	*W*/*B*	Unit weight (kg/m^3^ )										*AD* ^6^ (kg)	*AF* ^7^ (kg)
		*W*	*C*	*F*	*EA*	*SRA*	Zr	Steel Fibers ^1^	Steel Fibers ^2^	*S* ^3^ (Q) ^4^	*G* ^5^		
NSC	0.4	188	467	-	-	-	-	-	-	746	907	0.09	-
UHPC	0.14	178	783	235	59	8	196	39	78	(862)	-	26.1	0.78

^1^ 16.3 mm; ^2^ 19.5 mm; ^3^ Sand; ^4^ Quartz sand; ^5^ Gravel; ^6^ Admixture; ^7^ Anti-foaming agent.

**Table 7:** Results of compressive strength tests.

Division	Flowability (mm)	Curing		Compressive Strength (MPa)			Average Compressive Strength (MPa)
		Condition	Period (day)	1 (min)	2	3 (max)	
NSC	Slump 163 ± 10	Steam curing ^1^	7	25.8	26.1	26.7	26.2
		Air ^2^	7	13.3	13.8	14.1	13.7
			28	24.8	25.3	25.9	25.3
UHPC	Slump flow 680 ± 50	Steam curing	7	170.1	175.8	177.1	174.3
		Air	7	103.4	105.7	109.8	106.3
			28	126.5	127.5	128.3	127.4

^1^ High-temperature steam curing; ^2^ air dry curing.

**Table 8:** Results of bonding shear strength tests.

No.	Specimen Name	Delay	Maximum Load (kN)			Average Maximum Load (kN)	Shear Bond Strength (MPa)	Residual Shear Bond Strength Ratio (%)
			1	2	3			
1	NN-0	0	250.3	262.3	241.1	251.2	5.6	100
2	NN-15	15	228.9	229.9	239.3	232.7	5.2	92.4
3	NN-30	30	212.3	210.3	224.3	215.6	4.8	85.8
4	NN-60	60	198.1	194.9	176.5	189.8	4.2	75.6
5	TN-15	15	237.3	236.3	253.7	242.4	5.4	96.5
6	TN-30	30	240.8	233.8	236.2	236.9	5.3	94.3
7	TN-60	60	233.1	227.5	217.0	225.9	5.0	89.9
8	NU-0	0	1786.3	1708.7	1759.3	1751.4	38.9	100
9	NU-15	15	1504.1	1523.3	1445.8	1491.1	33.1	85.1
10	NU-30	30	1294.2	1386.1	1361.4	1347.2	29.9	76.9
11	NU-60	60	1026.0	1165.3	1132.3	1107.9	24.6	63.3
12	TU-15	15	1687.7	1664.5	1502.9	1618.4	36.0	92.4
13	TU-30	30	1418.5	1455.9	1319.9	1398.1	31.1	79.8
14	TU-60	60	1222.0	1263.7	1310.0	1265.2	28.1	72.2

**Table 9:** Failure modes of specimens.

Division	Tamping Condition	Failure Modes of Specimens			
		0 min	15 min	30 min	60 min
NSC	Non-tamping	N ^1^	N	I	I ^2^
	Tamping	N	N	N	N
UHPC	Non-tamping	N	I	I	I
	Tamping	N	N	I	I

^1^ Non-interfacial failure; ^2^ Interfacial failure.
